# Pro-resolving and anti-arthritic properties of the MC_1_ selective agonist PL8177

**DOI:** 10.3389/fimmu.2022.1078678

**Published:** 2022-11-24

**Authors:** Jose Garrido-Mesa, Bethan Lynne Thomas, John Dodd, Carl Spana, Mauro Perretti, Trinidad Montero-Melendez

**Affiliations:** ^1^ The William Harvey Research Institute, Queen Mary University of London, London, United Kingdom; ^2^ Palatin Technologies, Inc., Cranbury, NJ, United States; ^3^ Centre for inflammation and Therapeutic Innovation, Queen Mary University of London, London, United Kingdom

**Keywords:** melanocortin receptors, resolution pharmacology, inflammation, macrophage, MC1 receptor

## Abstract

**Background:**

Melanocortins are peptides endowed with anti-inflammatory and pro-resolving activities. Many of these effects are mediated by the Melanocortin receptor 1 (MC_1_) as reported in several experimental settings. As such, MC_1_ can be a viable target for the development of new therapies that mimic endogenous pro-resolving mediators. The aim of this study was to assess the immunopharmacology of a selective MC_1_ agonist (PL8177) *in vitro* and in a mouse model of inflammatory arthritis.

**Methods:**

PL8177 and the natural agonist αMSH were tested for activation of mouse and human Melanocortin receptors (MC_1,3,4,5_), monitoring cAMP accumulation and ERK1/2 phosphorylation, using transiently transfected HEK293A cells. The anti-inflammatory and pro-resolving effects of PL8177 and αMSH were evaluated using mouse peritoneal Macrophages. Finally, a model of K/BxN serum transfer induced arthritis was used to determine the *in vivo* potential of PL8177.

**Results:**

PL8177 activates mouse and human MC_1_ with apparent EC_50_ values of 0.01 and 1.49 nM, respectively, using the cAMP accumulation assay. Similar profiles were observed for the induction of ERK phosphorylation (EC_50_: 0.05 and 1.39 nM). PL8177 displays pro-resolving activity (enhanced Macrophage efferocytosis) and counteracts the inflammatory profile of zymosan-stimulated macrophages, reducing the release of IL-1β, IL-6, TNF-α and CCL-2. In the context of joint inflammation, PL8177 (3mg/kg i.p.) reduces clinical score, paw swelling and incidence of severe disease as well as the recruitment of immune cells into the arthritic joint.

**Conclusion:**

These results demonstrate that the MC_1_ agonism with PL8177 affords therapeutic effects in inflammatory conditions including arthritis.

**Significance:**

Drugs targeting the Melanocortin system have emerged as promising therapeutics for several conditions including inflammation or obesity. Multiple candidates are under clinical development, and some have already reached approval. Here we present the characterization of a novel drug candidate, PL8177, selective for the Melanocortin 1 receptor (MC_1_), demonstrating its selectivity profile on cAMP and ERK1/2 phosphorylation signaling pathways, of relevance as selective drugs will translate into lesser off-target effect. PL8177 also demonstrated, not only anti-inflammatory activity, but pro-resolving actions due to its ability to enhance efferocytosis (i.e. the phagocytosis of apoptotic cells), endowing this molecule with therapeutic advantages compared to classical anti-inflammatory drugs. Using a mouse model of inflammatory arthritis, the compound demonstrated *in vivo* efficacy by reducing clinical score, paw swelling and overall disease severity. Taken together, these results present Melanocortin-based therapies, and specifically targeting MC_1_ receptor, as a promising strategy to manage chronic inflammatory diseases.

## Introduction

Definition of mechanisms and mediators of the resolution of inflammation can guide the development of therapies that mimic the way our own body terminates this response (1). Multiple pro-resolving molecular mediators have been discovered and are currently in translational studies and drug discovery programs for the treatment of pathologies with an inflammatory component (2). The Melanocortin (MC) system constitutes one of these endogenous pro-resolving pathways. All natural Melanocortin agonists derive from the same proopiomelanocortin (POMC) protein, further cleaved into ACTH and the smaller melanocyte stimulating hormones (α, β, γMSH). ACTH was the first agonist studied and shown to be effective for the treatment of patients affected by rheumatoid arthritis (RA) (3). Melanocortins act on Melanocortin receptors (MC_1-5_), G-protein coupled receptors (GPCRs) that regulate multiple functions, such as skin pigmentation (MC_1_), steroidogenesis (MC_2_), energy homeostasis (MC_3,4_) or sebaceous gland function (MC_5_) (4). The high degree of similarity among these GPCRs makes challenging to achieve receptor selectivity (5). Although ACTH is the only MC agonist able to activate MC_2_-dependent steroidogenesis, regulatory functions on the inflammatory response can be achieved through the other MCRs (MC_1, 3-5_), independently of endogenous cortisol. Among them, MC_1_ stands out due to its wide distribution among the immune system and its influence on the inflammatory response.

MC_1_ activation reduces leucocyte recruitment and immune cell activation (6–13), Macrophage reactivity (14–16), promotes tolerogenic responses (17), and favors wound healing (18). The MC_1_ selective small molecule BMS-470539 has shown therapeutic efficacy in various models of neuro-inflammation (19, 20), ability to reduce leukocyte infiltration in a model of lung inflammation (16), improvement of membranous nephropathy (21) and reduction of joint inflammation using the K/BxN serum induced transfer arthritis (STIA) model (22). In addition, the MC_1_ selective peptide PL8177 can reduce experimental autoimmune uveitis (23) and intestinal inflammation in a model of inflammatory bowel disease (24). Data obtained from the use of mice lacking a functional MC_1_ receptor point to a major role for this receptor in regulating inflammation and maintaining homeostasis. For example, *Mc1r*-/- mice present with increased predisposition to vascular endothelial dysfunction (10), they develop more severe cartilage damage in experimental osteoarthritis (25) as well as more severe intestinal damage in an experimental model of colitis (26).

Of relevance for joint diseases, the Melanocortin system is also functional in non-immune cells, such as fibroblasts, osteoclasts, osteoblasts and chondrocytes (27–31). In fact, Melanocortin peptides have been detected in the synovial fluid of rheumatoid arthritis, osteoarthritis and juvenile chronic arthritis (32) and their levels negatively correlate with disease severity (33). Collectively, these reports highlight the relevance and therapeutic potential of MC_1_ and the Melanocortin pathway for the control of joint inflammation and tissue repair (27–31).

Disorders of the musculoskeletal system lead to chronic pain and disability that affects 19% of European population (34). Current pharmacological strategies may stop disease progression but are rarely able to induce healing (35, 36). We proposed that a fresh approach to the control of these diseases may be the development of agonists of endogenous protective mechanisms (2). However, among the major limitations of natural melanocortins is their lack of selectivity and an unfavorable pharmacokinetics (37). Therefore, the development of more selective and stable Melanocortin analogues may lead to novel therapeutics with improved translational potential for the treatment of joint inflammation (38) as well as other conditions.

Herein, we evaluated the pharmacological profile of PL8177, a synthetic cyclic heptapeptide selective for MC_1_ (24, 39). PL8177 has previously shown an interesting therapeutic potential in *in vivo* models of intestinal and ocular inflammation (24). In this study we investigated the signaling pathways engaged by PL8177 in human and mouse MC receptors and the post-receptor downstream functional outcomes using mouse primary peritoneal macrophages, known to express Melanocortin receptors (40). We also established the potential of PL8177 in a model of arthritis that recapitulates some features of active rheumatoid arthritis.

## Materials and methods

### Chemical compounds and preparation

PL8177 (provided by Palatin Technologies Inc) and αMSH (Tocris, Bristol, UK) solutions were prepared at 1mM stocks in DMSO (for *in vitro* studies) or PBS (*in vivo* studies) and single-use aliquots were frozen at -20°C. All other chemicals were obtained from Sigma-Aldrich, Poole, UK, unless otherwise indicated.

### Radioligand binding assay

B16-F1 melanocyte cell line naturally expressing MC_1_ were maintained in RPMI containing 10% fetal calf serum (FCS), 2 mM L-glutamine, 100 U/ml penicillin, and 100 μg/ml streptomycin and kept at 37°C with 5% CO_2_. Confluent monolayers were washed with PBS, harvested by gentle scrapping and centrifuged at 600 x g for 10 min. Pellets were resuspended in harvesting buffer and incubated with radioligand [^3^H]-PL8177 at a range of 1x10^-6^M to 1x10^-13^M for 90 min at room temperature. The endogenous Melanocortin agonist peptide αMSH was used as control. Binding was detected by scintillation counting and results are expressed as a percent of control specific binding and as a percent inhibition of control specific binding obtained in the presence of the test compound, PL8177. The inhibition constants (K_i_) were calculated using the Cheng Prusoff equation.

### Isolation of pCMV6-MCR plasmid constructs

Vectors for human and mouse MC_1_, MC_3_, MC_4_ and MC_5_ and empty vector pCMV6 were originally purchased from Origene (Rockville, Maryland, USA) and in-house transformed into bacteria. Bacterial clones were grown overnight in 150ml LB medium supplemented with kanamycin (25µg/ml). Plasmid DNA isolation was performed using Zyppy™ Plasmid Maxiprep kit (Zymo Research; Irvine, California, USA).

### Cell culture and transfection

HEK293A cells were maintained in DMEM containing 10% fetal calf serum (FCS), 2 mM L-glutamine, 100 U/ml penicillin, and 100 μg/ml streptomycin and kept at 37°C with 5% CO_2_. Cells were seeded in 96-well plates at 2x10^4^ cells/well and transfected 24 hours later with 50ng of plasmid DNA (encoding for human and mouse MC_1_, MC_3_, MC_4_ and MC_5_, TrueORF cDNA clones (Origene) and Lipofectamine 2000 (Invitrogen; Waltham, Massachusetts, USA) according to manufacturer’s instructions. Cells were used 24 h later.

### cAMP accumulation assay

Twenty-four hours after transfection, cells were serum-starved for 3 hours to reduce and stabilize basal levels of cAMP. Melanocortin agonists were tested using 1/5 serial dilutions to generate concentration response curves starting at 10 or 0.4 µM. Vehicle was used as negative control, and forskolin (3 µM, Tocris Bioscience) as positive control. Compound solutions were prepared in serum-free DMEM containing 10 mM 3-isobutyl-1-methylxanthine (IBMX; Sigma), to inhibit phosphodiesterase activity. Cells were stimulated for 15 min with the respective compounds and lysed immediately with 0.1M HCl followed by scrapping and freezing at -80°C until the assay was performed. cAMP was quantified using the Cyclic AMP Select ELISA kit (Cayman Chemicals, CAY501040, Ann Arbor, Michigan, USA) according to manufacturer instructions. Optical Density (OD) measurements were converted to cAMP concentration using a standard curve and results were normalized subtracting background signal and calculated as % of forskolin. The endogenous agonist αMSH (10 µM) was used to define the 100% agonistic effect for each receptor subtype.

### ERK1/2 phosphorylation assay

Twenty-four hours after transfection, cells were serum-starved for 3 hours to reduce and stabilize basal levels of phospho-ERK1/2. Concentration response curves were generated by 1/5 serial dilutions as above. Drug solutions were prepared in serum-free DMEM. Cells were stimulated for 5 min with the different compounds and lysed immediately with ice-cold extraction buffer supplied in the ERK1/2 (pT202/Y204) SimpleStep ELISA Kit (Abcam ab176640; Cambridge, UK), according to manufacturer instructions. Total protein was also quantified by Bradford to normalize p-ERK1/2 response. Basal absorbance levels (vehicle treated cells) were subtracted form test samples and data expressed as percentage of 10 µM αMSH effect, normalized to 100%.

### Animals

All animal studies were approved by and performed under the guidelines of the Ethical Committee for the Use of Animals, Barts and The London School of Medicine and Home Office regulations (Guidance on the Operation of Animals, Scientific Procedures Act, 1986). C57BL/6J wild-type (WT) mice were purchased from Charles River Laboratories (UK). Male mice (7-8 weeks old; body weight ~30 g) were maintained on a standard chow pellet diet and had free access to water, with a 12-hour light-dark cycle.

### Isolation and stimulation of Biogel™ elicited peritoneal Macrophages

C57BL/6J male mice were injected with 1 ml of 2% Biogel intraperitoneally (Bio-Rad; Hercules, California, USA). Four days later, peritoneal cells were collected by lavage using 4 ml of 3mM EDTA in PBS and plated in RPMI 1640 containing 10% FCS. After 1 h of incubation of peritoneal lavage, wells were washed to remove non-adherent cells and experiment initiated. For cytokine production, the different conditions were tested in duplicate using 24-well plates with 0.5x10^6^ cells/well. For mRNA analyses, 2.5x10^6^ cells/well in 6-well plates were used. Compounds or vehicle were added 30 min before stimulation with 25 µg/ml zymosan A (Sigma-Aldrich) for 6 h. Then, supernatants and cells were collected for cytokines measurements and gene expression analyses, respectively.

### Quantification of gene expression by peritoneal Macrophages by RT-qPCR

Total RNA was extracted using RNAeasy Plus Mini Kit (Qiagen; Hilden, Germany) following manufacturer’s instructions. cDNA was synthesized using the SuperScript III VILO Master Mix (Invitrogen). Real time-PCR was performed in duplicates, with 4µl cDNA (20 ng), 1µl of primers and 5µl iTaq™ Universal SYBR^®^ Green PCR Master Mix (Applied Biosystems), using the ABI Prism 7900HT Sequence Detection System. Dissociation step was always included to confirm the absence of unspecific products. For relative gene expression, fold increase was calculated with the formula 2^(-ΔΔCt)^, using *Gapdh* as reference gene. Quantitect primers (QIAGEN) used were the following: *Alox15* (QT00111034), *Alox5* (QT00258622), *Ano6*, (QT01038660), *Anxa1* (QT00145915), *Cd14* (QT00246190), *Gapdh* (QT01658692), *Hmgb1* (QT00247786), *Hmox1* (QT00159915), *Il1b* (QT01048355), *Il6* (QT00098875), *Lgals3* (QT00152558), *Lgals9* (QT00173236), *Lta4h* (QT00160475), *Nos2* (QT00100275), *Pparg* (QT00100296), *Ptges* (QT00118223), *Tlr4* (QT00259042).

### Quantification of cytokine release by peritoneal Macrophages by ELISA

IL-6, TNF-α (both 1/10 dilution), IL-1β and CCL-2 (undiluted) were measured by ELISA (Invitrogen; Waltham, Massachusetts, USA). Concentration values were extrapolated from a standard curve and expressed as increase over basal cytokine levels for each individual donor mouse.

### Isolation of human primary neutrophils

Experiments using healthy volunteers (written consent provided) were approved by Queen Mary Ethics of Research Committee QMREC2014.61. Blood was collected into 3.2% sodium citrate (1:10) and diluted 1:1 in RPMI 1640 before separation through a double-density gradient using Histopaque 10771 and 11191 (Sigma-Aldrich). Contaminating erythrocytes were removed by hypotonic lysis. Polymorphonuclear cells were incubated in 10% FCS overnight at 37°C, 5% CO_2_ to let neutrophils undergo spontaneous apoptosis.

### Evaluation of Macrophage efferocytosis

Mouse primary peritoneal Macrophages (0.5x10^6^ cells/well in 24-well plates, in duplicate) were stimulated with compounds/vehicle for 30 min before the addition of apoptotic neutrophils (1:5 ratio, Macrophage:neutrophil) for 1 h. Cells were washed 3 times and fixed for 30 min with 2.5% glutaraldehyde and neutrophils stained using the myeloperoxidase assay by adding 0.1 mg/ml dimethoxybenzidine (Sigma-Aldrich) and 0.03% (v/v) hydrogen peroxide for 1 h. Cells were analysed by light microscopy. Ten random fields were acquired, and more than 400 cells were blindly counted per well. Clearance index was calculated as (% phagocytic cells x % multiple ingestions cells) x100.

### K/BxN serum transfer induced arthritis (STIA) model

Arthritis was induced in C57BL/6J male mice (10 weeks old) by two i.p. injections of 100μl of K/BxN serum (diluted 1:1 in PBS, i.e. 200μl volume injected) on days 0 and 2. Arthritic mice (n=24) were randomized in 4 cages, each one including 2 mice from each different experimental group (n=6 mice per cage). Compound PL8177 was prepared as a 0.1mg/ml solution in PBS and stored frozen in single use aliquots. From day 2 to 7, vehicle PBS or PL8177 (3 or 0.3 mg/kg) daily treatment was administered by 0.8ml i.p. injection. The development of systemic arthritis was monitored daily measuring clinical score, paw volume (plethysmometer) and knee width (caliper). To obtain the clinical score, each of the four limbs was evaluated for signs of inflammation in wrist/ankle, pad and digits (one point each of these 3 parts), thus reaching a maximum of 12 (3 per limb). Mice were sacrificed at day 7, and different tissue samples were collected for evaluation. An additional group of non-arthritic mice (n=6) was also included for baseline measurements.

### Flow cytometry analysis

For the evaluation of joint immune infiltration, cells were isolated from the hind paws by digestion in serum-free media containing DNAse I (40µg/ml) and collagenase D (0.5µg/ml) for 1 h. Upon tissue digestion, 30,000 precision counting beads (BioLegend; San Diego, California, USA) were added for total cell quantification and the cell suspension was incubated with live/dead discrimination marker (Fixable Viability eF780, Thermo Fisher Scientific; Waltham, Massachusetts, USA), Fc Receptor blocking reagent (BioLegend) and subsequently stained with an antibody cocktail to characterize the different immune cell populations. The antibodies used were from BioLegend, unless otherwise specified: Ly6C-BV421 (clone HK1.4), CD45-VioGreen (clone 30F11; Miltenyi; Bergisch Gladbach, Germany), MHC-II-BV650 (clone M5/114.15.2), CD11b-BV785 (clone M1/70), B220-FITC (clone RA3-6B2), F4/80-PE (clone BM8), CD3e-PE-Cy5 (clone 145-2C11), SiglecF-PE-Vio770 (clone REA798; Miltenyi), CD11c-APC (clone N418) and Ly6G-AF700 (clone 1A8). A sample containing at least 20,000 beads was acquired in a Fortessa Cytometer (BD; Franklin Lakes, Nueva Jersey, USA) and flow cytometry data was analyzed with FlowJo software v10.4 (ThreeStars).

### Data analysis

Non-linear regression models were used to generate dose–response curves. Statistical analysis was performed with GraphPad Prism v8 using either t-test (2 groups) or one-way ANOVA test (>2 groups), and paired tests for repeated measures when appropriate, followed by Tukey’s multiple comparison test. Values of p<0.05 were considered statistically significant.

## Results

### PL8177 selectively activates mouse and human MC_1_ receptors

**Figure 1:**
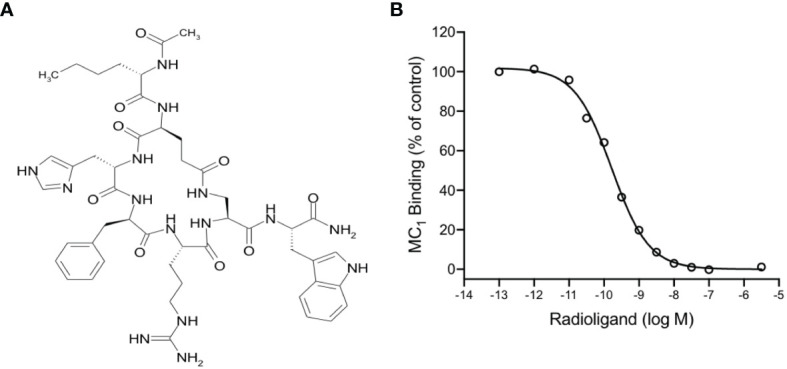
Chemical structure and binding of PL8177 to the mouse MC_1_ receptor **(A)** Structure of PL8177, with amino acid sequence Ac-Nle^1^-cyclo(Glu^2^-L-His^3^-D-Phe^4^-Arg^5^-Dap^6^)-Trp^7^-NH_2_. **(B)** Binding affinity of PL8177 (0.1 pM – 10 µM) for mouse MC_1_ receptor, expressed in B16-F1 cell line (Ki =75 pM). Data represent regression curve and mean values of n=3 independent measurements.

PL8177 is a synthetic analog derived from the sequence of the natural αMSH peptide. It is cyclized by an amide bond between glutamic and diaminopropionic acid side chains and includes one D-amino acid (D-phenylalanine) (**Figure 1A**). In binding affinity studies with mouse MC_1_ expressed in B16-F1 cell line, PL8177 displayed high affinity for this receptor (Ki =75 pM; **Figure 1B**), while the affinity for MC_3_ and MC_4_ was substantially weaker using transiently transfected HEK293 cells (3500nM and 360nM respectively -data not shown).

To characterize the signaling profile and selectivity of PL8177 for the different MC receptors, concentration-response curves were generated in human embryonic kidney cells, HEK293A, transiently transfected with mouse or human MC_1_, MC_3_, MC_4_ or MC_5_ receptors. Both cAMP accumulation and ERK1/2 phosphorylation were used as readouts to evaluate their agonistic activity.

**Figure 2:**
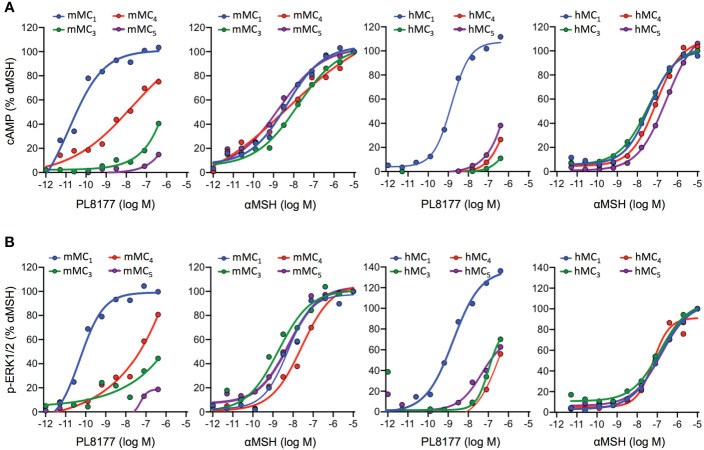
Melanocortin receptors signaling induced by PL8177 and αMSH. Concentration dependent accumulation of cAMP **(A)** and formation of ERK1/2 phosphorylation (p-ERK1/2) **(B)** upon agonist stimulation (15 min for cAMP; 5min for p-ERK1/2) of HEK293A cells transiently transfected with mouse or human Melanocortin (MC) receptors. Data are expressed as percentage of maximal αMSH response and represent mean values of n=3 independent experiments, each one performed in duplicate.

Quantification of cAMP accumulation (**Figure 2A**) showed that PL8177 peptide triggered MC_1_-mediated cAMP production at picomolar-nanomolar range (EC_50_ values of 0.01 and 1.49nM for mouse and human receptors respectively, see **Table 1**), while reduced or no significant effect was observed on cells transfected with MC_3,4,5_ receptors subtypes at those concentrations. By contrast, the natural agonist αMSH activated all MC receptor subtypes, both mouse and human, showing no receptor selectivity as expected (**Figure 2A** and **Table 1**). The potency at MC_1_ was lower than that obtained for PL8177, with EC_50_ values of 4.37 and 37.07nM for mouse and human MC_1_ respectively. A similar pattern was observed when phosphorylation of ERK1/2 was measured (**Figure 2B**): whilst αMSH induced ERK1/2 phosphorylation following activation of all four receptor subtypes, the synthetic peptide PL8177 displayed a strong selectivity for MC_1_ receptor, with EC_50_ values of 0.05nM and 1.39nM for mouse and human receptors respectively. On the other MC receptors, PL8177 had only partial or null phospho-ERK1/2 activity (**Figure 2B** and **Table 1**).

**Table 1:** Human and mouse Melanocortin receptor signaling parameters of PL8177 and αMSH.

	mMC_1_		mMC_3_		mMC_4_		mMC_5_		hMC_1_		hMC_3_		hMC_4_		hMC_5_	
	αMSH	PL8177	αMSH	PL8177	αMSH	PL8177	αMSH	PL8177	αMSH	PL8177	αMSH	PL8177	αMSH	PL8177	αMSH	PL8177
**A) cAMP**																
**Top**	96.18	103.48	86.21	40.54	78.75	75.09	97.08	14.85	85.85	111.69	83.66	10.92	79.02	26.54	65.94	38.25
**Top αMSH/Top PL8177**	1.00	1.08	1.00	0.47	1.00	0.95	1.00	0.15	1.00	1.30	1.00	0.13	1.00	0.34	1.00	0.58
**Hill Slope**	0.49	0.50	0.42	0.49	0.19	0.28	0.40	0.62	0.67	0.93	0.60	1.13	0.67	0.95	0.60	0.60
**EC**_50_ (nM)	4.37	0.01	16.06	~ 7.34+E07	5.05	20.52	1.96	~ 1.07+E06	37.07	1.49	36.31	597.04	84.92	744.73	256.45	1.56E+05
**R²**	0.93	0.93	0.88	0.78	0.95	0.95	0.96	0.42	0.90	0.97	0.91	0.94	0.97	0.91	0.96	0.99
**B) p-ERK1/2**																
**Top**	95.43	99.69	103.84	44.39	94.10	80.74	97.29	18.65	70.52	136.15	70.94	70.08	86.53	56.08	70.19	62.66
**Top αMSH/Top PL8177**	1.00	1.04	1.00	0.43	1.00	0.86	1.00	0.19	1.00	1.93	1.00	0.99	1.00	0.65	1.00	0.89
**Hill Slope**	0.72	0.71	0.55	0.22	0.58	~ 0.2002	0.57	~ 11.96	0.52	0.63	0.60	1.27	0.88	0.71	0.59	0.77
**EC_50_ (nM)**	5.67	0.05	1.84	~ 2.08+E11	27.05	~ 9.41+E11	6.94	~ 72.94	116.40	1.39	159.96	104.47	64.12	592.93	174.58	39.63
**R²**	0.91	0.85	0.87	0.46	0.86	0.77	0.85	0.36	0.99	0.90	0.91	0.63	0.88	0.86	0.91	0.78

Pharmacological parameters of concentration-response curves corresponding to the data presented in **Figure 2** for cAMP accumulation (A) and formation of ERK1/2 phosphorylation (p-ERK1/2) (B) upon Melanocortin stimulation of HEK293A cells transiently transfected with mouse or human Melanocortin (MC) receptors. Due to the scarce effect of PL8177 on MC_3,4,5_, the calculated parameters are reported as ambiguous values (indicated with the symbol ~). ‘Top’ refers to normalised maximal efficacy at 2μM; “Top PL8177/Top aMSH” is the ratio between the maximal efficacy at 2μM between PL7188 and αMSH, indicative of full (~1) or partial agonism (<1).

Altogether, the results obtained suggest that the pharmacological activity of PL8177 corresponds to a selective MC_1_ agonist with a non-biased profile with respect to cAMP and ERK, and improved potency compared to the endogenous agonist αMSH.

### MC_1_ agonism with PL8177 exerts anti-inflammatory and pro-resolving properties on mouse primary Macrophages *in vitro*

**Figure 3:**
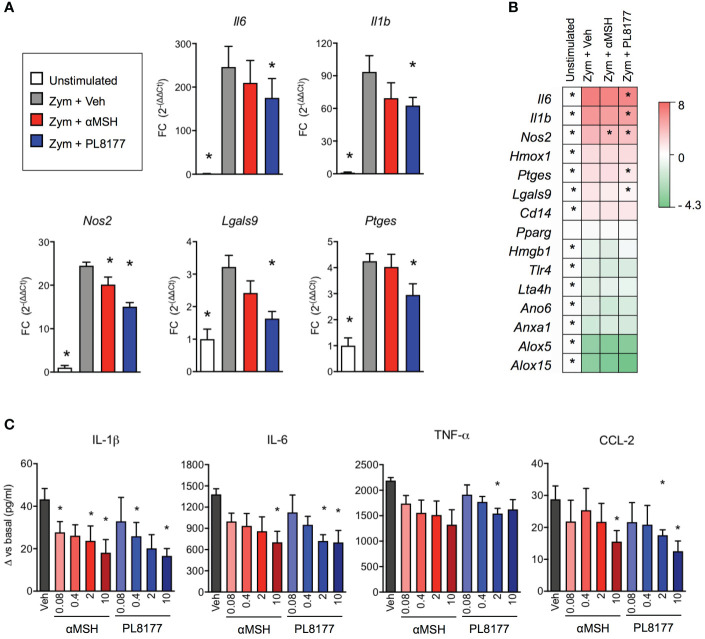
*In vitro* anti-inflammatory actions of PL8177 and αMSH on mouse primary macrophages. Biogel-elicited peritoneal macrophages were incubated for 30 min with Melanocortin compounds prior stimulation with zymosan (Zym, 25μg/ml) for further 6 h. **(A)** Gene expression for inflammatory related genes was measured by real time-PCR on cells treated with vehicle (Veh) or with 2μM of PL8177 or αMSH. Unstimulated cells were used as control for normalization. Fold changes (FC) were calculated as 2^(-ΔΔCt)^, using *Gapdh* as reference gene **(B)** Summary of gene expression measurements, including genes that were not significantly altered by the treatments, expressed as log_2_FC. **(C)** Concentration-response effect of PL8177 and αMSH (0.08-10μM) on cytokine release by macrophages measured by ELISA. For all experiments, 5 mice were used and analyzed by repeated measures one-way ANOVA followed by multiple comparison test. Data represent mean ± SEM. *p<0.05 *vs.* zymosan alone (Zym).

Treatment of biogel-elicited peritoneal macrophages with Melanocortin peptides (2 µM) for 30 minutes was followed by a 6-hour incubation period with zymosan (25 μg/ml). This inflammogen produced a classical activation of inflammatory genes like interleukins 1 beta and 6 (*Il1b, Il6)*, inducible nitric oxide synthase (*Nos2*), prostaglandin E synthase (*Ptges*), galectins 3 and 9 (*Lgals3, Lgals9*), heme-oxygenase 1 (*Hmox1*) and the LPS co-receptor *Cd14* (**Figures 3A, B**). On the other hand, gene expression levels were reduced for annexin A1 (*Anxa1*), anoctamin 6 (*Ano6*), high motility group box 1 (*Hmgb1*), lipoxygenases 5 and 15 (*Alox5, Alox15*), leukotriene A4 hydrolase *(Lta4h*) and toll-like receptor 4 (*Tlr4*). Both PL8177 and αMSH modulated the response to zymosan in a selective manner, with reduction in the expression of *Il1b*, *Il6*, *Nos2*, *Lgals9* and *Ptges* (**Figures 3A, B**), without reverting the effect on anti-inflammatory genes, at least at this 6-hour time-point. These effects were mirrored by a partial modulation of cytokine levels released in the supernatants (**Figure 3C**). Herein, PL8177 and αMSH were tested at 80 nM-10 µM range, with more prominent reductions (~60%) on IL-1β, IL-6 and CCL-2 release.

**Figure 4:**
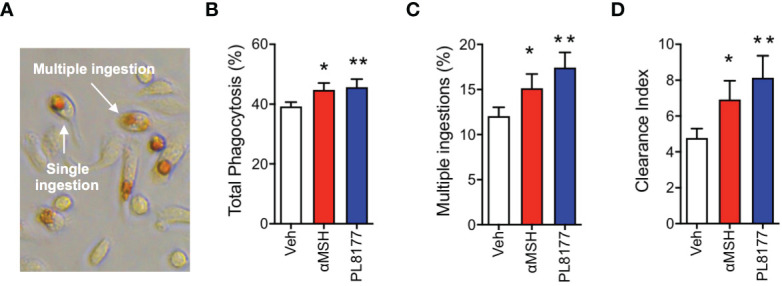
Pro-resolving effects of PL8177 on Macrophage efferocytosis. Biogel-elicited peritoneal macrophages were incubated with Melanocortin compounds (2μM) for 30 min prior to the addition of apoptotic neutrophils (at a Macrophage:neutrophil ratio of 1:5). After 1 h, cells were stained using the myeloperoxidase (MPO) assay to visualize ingested neutrophils. **(A)** Representative image shows the dark brown coloration selectively acquired by engulfed neutrophils. An example of single and a multiple ingestion are indicated. **(B)** Total phagocytosis was calculated as % of macrophages containing at least one neutrophil inside. **(C)** Multi-phagocytic cells containing more than 1 neutrophil were also quantified. These data show the percentage of macrophages that have ingested more than one neutrophil, with respect to the total Macrophage population **(D)** Clearance Index was calculated as (% phagocytic cells x % multiple ingestions) x100. Data represent mean ± SEM of 5 mice analyzed by repeated measures one-way ANOVA followed by multiple comparison test. *p<0.05, **p<0.01 vs. untreated (Veh).

To complete the characterization of PL8177 as a genuine pro-resolving compound, we tested the ability of this peptide to enhance Macrophage efferocytosis (i.e. the phagocytosis of apoptotic cells), using apoptotic neutrophils to feed the macrophages and quantifying efferocytosis by performing the myeloperoxidase assay (**Figure 4A**). At 2 µM concentration, PL8177 augmented the already marked efferocytosis (~16% increase over basal levels, **Figure 4B**) as well as the presence of multi-phagocytic macrophages (i.e. cells that have engulfed more than one apoptotic neutrophil, **Figure 4C**). Such properties of PL8177 yielded a highly significant effect on the clearance index (**Figure 4D**).

### The MC_1_ agonist PL8177 reduces inflammation in a mouse model of arthritis

**Figure 5:**
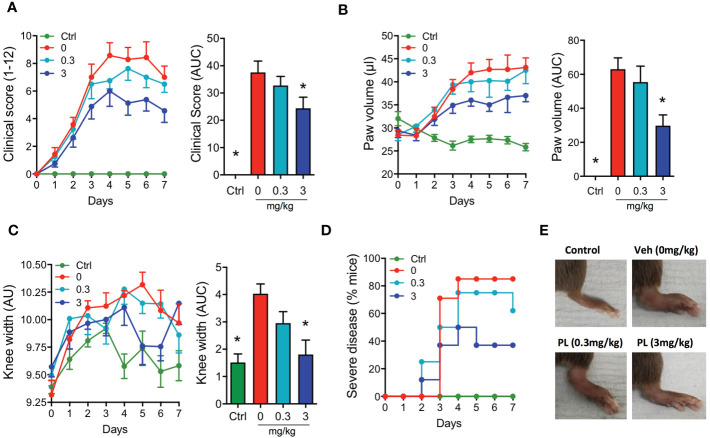
Effects of PL8177 in the K/BxN serum transfer induced arthritis (STIA) model Arthritis was induced by injecting mice with 100μl of K/BxN serum at days 0 and 2. A group of mice that did not receive serum was used as control (Ctrl). Mice were treated daily with vehicle (0mg/kg) or with PL8177 (0.3 or 3mg/kg) from day 2 to 7. Daily measurements of clinical score **(A)**, paw volume **(B)** and knee width **(C)** are shown, together with the quantification of the area under the curve (AUC) calculated from days 2-7, corresponding to the duration of the treatments (bar graphs). **(D)** The percentage of mice developing moderate-severe arthritis (score ≥ 6) is presented. Representative images of the hind paws at day 7 are shown in **(E)** Data represent mean ± SEM of n=6-8 mice analysed by one-way ANOVA followed by multiple comparison test. *p<0.05 vs. vehicle group (0mg/kg).

Injection of K/BxN serum to mice (day 0 and 2) promoted a rapid onset of polyarthritis with parameters elevated from day 4 onwards, including clinical score, paw oedema and knee width (**Figures 5A–C**). Drug treatment started at day 2, with animals being randomized to the distinct treatment groups. PL8177 was tested at two doses (0.3 and 3mg/kg), with the higher one significantly attenuating clinical score (-35%; **Figure 5A**), the development of oedema (**Figure 5B**), the incidence of severe disease (**Figure 5D**), and to a lesser extent, knee swelling (**Figure 5C**). Representative images of paws collected at the end of the study show evident signs of swelling and inflammation in vehicle-treated arthritic mice and improvement with the PL8177 treatment (**Figure 5E**). The lower dose of PL8177 (0.3mg/kg) offered a degree of improvement on the parameters under analyses in the STIA model, but none of them reached statistical significance (**Figure 5**).

**Figure 6:**
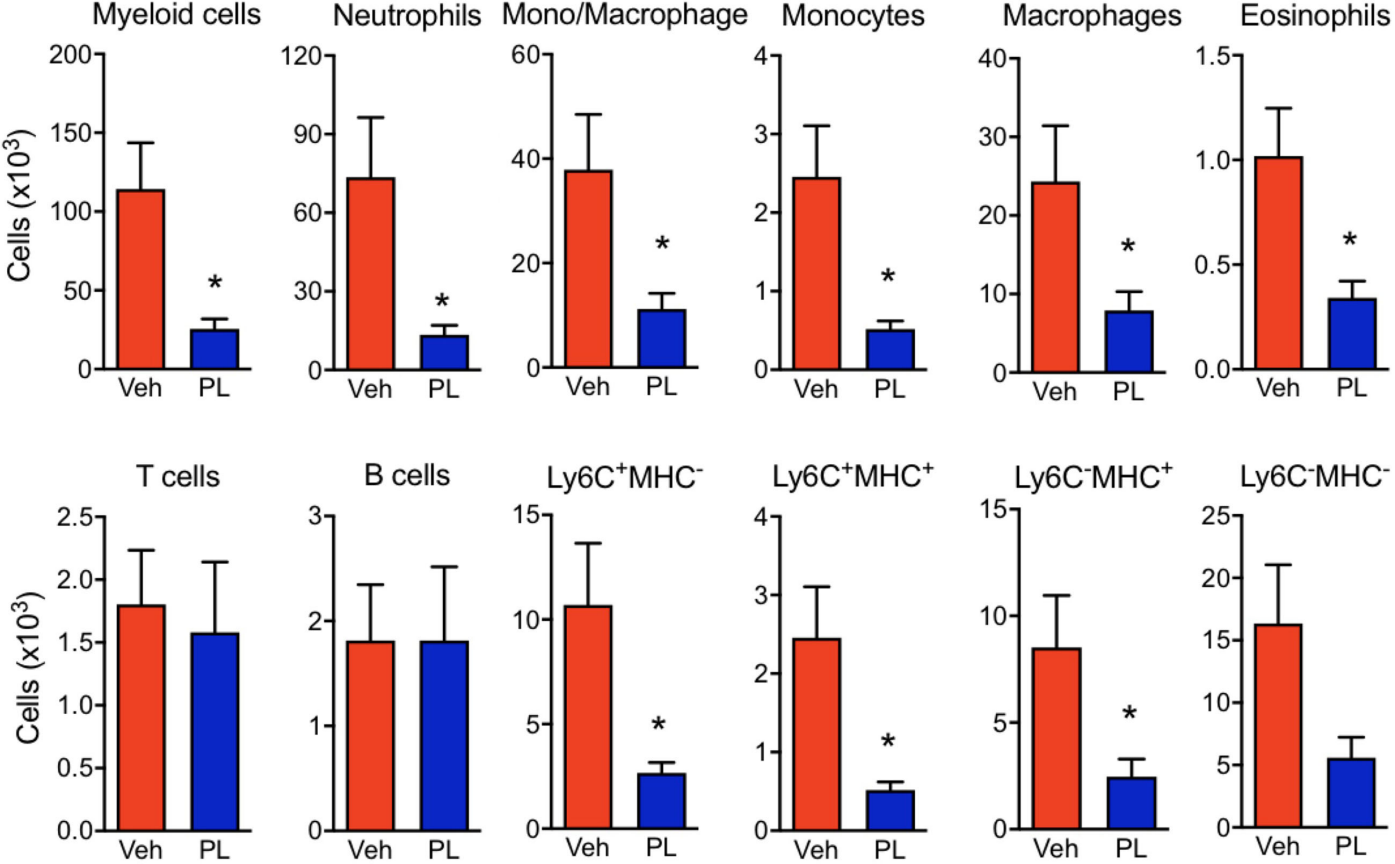
Effect of PL8177 in immune cell recruitment to arthritic joints. Flow cytometry analysis of hind paw cells of arthritic vehicle-treated (Veh) and PL8177-treated (3 mg/kg) mice was performed. Total cell numbers were obtained by normalization with quantification beads added upon enzymatic digestion. The gating strategy is provided in **Supplementary Figure 1**.The different populations were defined as follows: total myeloid cells (CD45^+^ CD11b^+^), neutrophils (CD45^+^ CD11b^+^ Ly6G^+^), mono-macrophages (CD45^+^ CD11b^+^ SiglecF^-^ Ly6G^-^), monocytes (CD45^+^ CD11b^+^ SiglecF^-^ Ly6G^hi^ F4/80^lo^), macrophages (CD45^+^ CD11b^+^ SiglecF^-^ Ly6G^lo^ F4/80^hi^), eosinophils (CD45^+^ CD11b^+^ SiglecF^+^), T cells (CD45^+^ CD11b^-^ B220^-^ CD3e^+^), B cells (CD45^+^ CD11b^-^ B220^+^). The mono/Macrophage population was also analysed based on Ly6C and MHC-II markers. Data are mean ± SEM of n=7 analyzed by t-test. *p<0.05 vs. vehicle-treated arthritic mice (Veh).

The effects of PL8177 on the macroscopic signs of arthritis were matched by a modulation of immune cell phenotype in the arthritic joints (**Figure 6** and **Supplementary Figure 1**). At day 7, the paw immune infiltrate was mostly myeloid, with ~65% of cells being neutrophils (CD11b^+^Ly6G^+^), ~2% monocytes (CD11b^+^Ly6C^+^) and ~20% macrophages (CD11b^+^F4/80^+^). At the dose of 3 mg/kg, PL8177 did not affect the small number of B cells (B220^+^) and T cells (CD3e^+^), while producing a marked reduction in myeloid cell infiltration (**Figure 6**). The latter effect was due to significant differences in the number of neutrophils (-81%), eosinophils (CD11b^+^SingleF^+^) (-66%) and monocytes-macrophages (-70%). Further analyses of the different subsets of monocytes and macrophages using the combination of Ly6C, F4/80 and MHC-II markers, revealed a slightly higher inhibitory effect in the abundance of the immature monocyte populations Ly6C^+^/MCH-II^-^ and Ly6C^+^/MCH-II^+^ cells.

## Discussion

In this study, we provide evidence that the Melanocortin agonist PL8177 possesses anti-inflammatory and pro-resolving properties that translated into an amelioration of experimental arthritis *in vivo*. Such protective actions are likely downstream the selective activation of MC_1_, as shown with receptor signaling experiments using both human and mouse Melanocortin receptors. In addition to selectivity, another improvement that this peptide achieves compared to the endogenous αMSH is the increased potency, with EC_50_ values 25-fold (cAMP) and 84-fold (p-ERK1/2) lower for PL8177 compared to αMSH, at the human MC_1_. Therefore, PL8177 can be proposed as a viable therapeutic tool to modulate inflammatory diseases including arthritis.

Melanocortin-based pro-resolving mediators are of growing interest for research and pharmaceutical industry (36) and selective MC_1_ agonists may represent ideal candidates for potential therapeutic development. The pan-agonist afamelanotide ([Nle^4^,D-Phe^7^]-αMSH) (41) is approved by the European and U.S. regulatory administrations for the treatment of phototoxicity associated with erythropoietic protoporphyria (42), yet it is not selective and may potentially produce undesired side effects altering the other physiological roles of MC_3-5_ receptors. Natural Melanocortin agonists are metabolically unstable and are rapidly degraded, with half-lives of ~20 min for αMSH (43), a major hurdle in the development of drugs based on Melanocortin peptides (44–47). PL8177 is designed as a cyclic structure to improve stability (47) and, indeed, pharmacokinetic characterization has shown persisting plasma levels 48h after a single subcutaneous administration in humans, with a half-life of 12.6 ± 3.8 h after 7 days of 3 mg multiple-dosing (39). Thus, PL8177 has superior pharmacokinetic properties compared to other synthetic Melanocortin agonists currently in development (16, 48), in addition to the improved selectivity and potency profiles that we show here.

Our study began by assessing the effect of PL8177 on both mouse and human Melanocortin receptors. Over a broad concentration-range, PL8177 displayed agonistic activities selectively for MC_1_, with little effect on the other members of the receptor family. In these assays, we tested both cAMP accumulation and phosphorylation of ERK1/2, since both signaling pathways operate downstream of this family of G-protein coupled receptors (40). In this set of experiments, PL8177 did not show any preference for one signaling pathway and as such was pharmacologically distinct from the small molecule AP1189, another Melanocortin compound under clinical development which we previously reported to be a biased agonist towards phospho-ERK1/2 pathway (49). It would be of interest to compare the effects of PL8177 and AP1189 in settings of chronic disease, to establish the potential difference in beneficial properties for a highly selective agonist (PL8177) versus a non-selective yet biased agonist (AP1189).

PL8177 has been reported to modulate LPS-induced TNF-α production using human whole blood stimulation (24). In our *in vitro* assays with mouse macrophages, PL8177 provided an interesting profile with a preferential modulation of pro-inflammatory gene expression rather than a reversal of anti-inflammatory genes. This is quite novel and could be linked to the time-point used, 6-hour stimulation, which could still be in the onset phase of cell activation. In any case, these data add to the well-known properties of the MC_1_ receptor on Macrophage response (14–16, 50). In this context, the effect of PL8177 on efferocytosis was also of interest, as this activity was originally identified to endow Melanocortin agonists with genuine pro-resolving properties (40, 50, 51). It was intriguing to identify a remarkable effect of PL8177 on the clearance index and on the phagocytic ability of the Macrophage at the single cell level (i.e. the ability for multiple ingestions). Future studies may detail the mechanistic implications of the observed effect and clarify what may be unique for the pool of macrophages that responded to the selective MC_1_ agonist. One could determine potential association with efferocytic receptors like MERT-K or receptors for the acidic phospholipids phosphatidyl serine. Another interesting aspect for future studies is the investigation of the potential differential responses that PL8177 may have in M1 and M2 macrophages, and if this could be harnessed therapeutically. In the above signaling experiments, weak partial agonistic activity was observed at other MCRs which have been reported to elicit anti-inflammatory activities, like MC_3_ and MC_4_. Although it is unlikely that the actions of PL8177 are substantially mediated by these other receptors, it would be interesting to assess the actions of this peptide in mice lacking the receptor, to fully demonstrate that the actions are solely mediated by MC_1_.

Quantification of cytokine levels on the Macrophage supernatants revealed that PL8177 afforded the expected reduction on cytokine release, albeit with a modulatory effect, that is an inhibition in the 30-40% range. This is in line with our previous studies using biogel-elicited peritoneal macrophages, where we tested αMSH, [D-Trp^8^]-γMSH, [Nle^4^,D-Phe^7^]-αMSH, the small molecule BMS-470539 and the pan-agonist AP214 (40, 50). Equally in agreement with these previous studies, reduction in IL-1β was the outcome mostly affected by PL8177. An important modulation of IL-6 release was also observed after cell incubation with PL8177. A more prominent effect of the selective MC_1_ agonist on these two cytokines prompted the next series of experiments. Here we focused on inflammatory arthritis, since both IL-1β and IL-6 are central in this pathology. In fact, the K/BxN STIA model of inflammatory arthritis is highly reliant on IL-1β as shown using genetic and pharmacological approaches (52, 53). Similarly, IL-6 is a major pro-arthritic cytokine that connects pathogenic mechanisms between immune cells and synovial fibroblasts (54–56). Whilst the role of IL-6 may be secondary compared to IL-1β for the development of pathology in the K/BxN STIA model, its inhibition leads to therapeutic benefit in experimental and human rheumatoid arthritis, as both cytokines are key therapeutic targets to stop disease progression (57).

We have reported the role of the Melanocortin system in joint inflammation, proposing a major effect through the targeting of immune cells. However, the MC_1_ receptor is prevalent in other cells within the joint tissue, including on synovial fibroblasts (58) and chondrocytes (28, 29). So, altogether there is scope to establish the potential protective properties of PL8177 in inflammatory arthritis. K/BxN STIA is a mouse model ideal for anti-arthritic drug testing, with its fast onset (38), and with an inflammatory reaction initiated by complement fixation onto cartilage, thus highly reliant on the innate immunity, and the engagement of cells like monocytes, macrophages and neutrophils (59). Therapeutic administration of PL8177 reduced disease progression and development of severe pathology. The distal joint (wrist/ankle), where the inflammatory process had a higher impact compared to knees, was also the location where PL8177 treatment had a higher efficacy (≥50% reduction in oedema and myeloid cell recruitment). Under prevailing inflammatory conditions, tissue recruited neutrophils fail to undergo apoptosis, delaying inflammatory resolution. MC_1_ agonism has been shown to inhibit delayed neutrophil apoptosis (60). This mechanism and the improvement of Macrophage efferocytosis, reported here for PL8177, provide an MC_1_-regulated pathway towards improved neutrophil tissue clearance. This pro-resolving effect, together with diminished recruitment due to the anti-inflammatory actions of PL8177, could explain the marked reduction in neutrophils counts.

In the effector phase of human RA and in murine arthritis, IL-1β production by monocytes/macrophages contributes to disease pathogenesis due to chondrocyte activation and cartilage damage (61). Reduction of IL-1β was among the anti-inflammatory effects of PL8177 on mouse macrophages *in vitro*, followed by a modest reduction in CCL-2, a chemokine that promotes monocyte recruitment. Once monocytes enter the joint tissue, the local environment produces a shift towards an inflammatory phenotype, similar to the *in vitro* M1 activation profile (62). In our evaluation of the anti-inflammatory effects of PL8177, we observed a reduction of classical M1 markers, such as *Nos2*, *Il1b* and *Il6* expression, in part confirmed by measurement of cytokine release as discussed above. The translation of this effect *in vivo* would result in reduced inflammatory amplification, as observed with reduced oedema and innate immune recruitment in the STIA model.

In conclusion, these preclinical investigations describe the anti-inflammatory, pro-resolving and anti-arthritic effects of PL8177, a selective and potent MC_1_ agonist. PL8177 has already shown therapeutic benefit given intraperitoneally in a mouse model of autoimmune uveitis, as well as in intestinal inflammation in rats upon oral administration (24). This new study highlights the potential clinical utility of PL8177, or of MC_1_ selective agonists in general, adding inflammatory arthritis to the list of immune-inflammatory disorders that could be targeted using a Melanocortin-based therapeutic approach.

## Data availability statement

The raw data supporting the conclusions of this article will be made available by the authors, without undue reservation.

## Ethics statement

Experiments using healthy volunteers (written consent provided) were approved by Queen Mary Ethics of Research Committee QMREC2014.61. The patients/participants provided their written informed consent to participate in this study. All animal studies were approved by and performed under the guidelines of the Ethical Committee for the Use of Animals, Barts and The London School of Medicine and Home Office regulations (Guidance on the Operation of Animals, Scientific Procedures Act, 1986).

## Author contributions

Conceptualization: TM-M, MP, JD, CS. Methodology: JG-M, BT. Analysis: JG-M, BT, Data interpretation: JG-M, TM-M, MP. Visualization: JG-M. Manuscript writing and review: JG-M, TM-M, MP. Funding acquisition: MP, T-MM; Supervision: TM-M, MP.

## Funding

This project was funded by Palatin Technologies Inc, through a collaborative agreement with William Harvey Research Limited. The funder was not involved in the study design, collection, analysis, interpretation of data, the writing of this article or the decision to submit it for publication.

## Conflict of interest

JD, CS are employees of Palatin Technologies, Inc. MP has received consultancy fees from Palatin Technologies, Inc.

The remaining authors declare that the research was conducted in the absence of any commercial or financial relationships that could be constructed as a potential conflict of interest.

## Publisher’s note

All claims expressed in this article are solely those of the authors and do not necessarily represent those of their affiliated organizations, or those of the publisher, the editors and the reviewers. Any product that may be evaluated in this article, or claim that may be made by its manufacturer, is not guaranteed or endorsed by the publisher.

## Supplementary material

Supplementary Figure 1 Gating strategy for the analysis of hind paw immune cell infiltration. Representative flow cytometry analysis following a sequential gating strategy. Populations of interest are annotated with numbers (populations P1-P19). Recorded events were cleaned to select single events based on forward scatter FSC (P1) and side scatter SSC (P2) signals, and according to cell size (P3). Counting beads (P4) were gated using their autofluorescence and live cells (P5) were selected based on live/dead (L/D) discrimination marker. Live CD45^+^ immune cells (P6) were categorised in 3 main populations according to the expression of the following markers: B cells (P7: CD45^+^ CD11b^-^ B220^+^), T cells (P9: CD3e^+^ cells selected from P8: CD45^+^ CD11b^-^ B220^-^) and myeloid cells (P10: CD45^+^ B220^-^ CD11b^+^). Eosinophils (P11: CD45^+^ CD11b^+^ SiglecF^+^) and neutrophils (P12: CD45^+^ CD11b^+^ Ly6G^+^) were selected among myeloid cells based on SiglecF and Ly6G markers, and the double negative myeloid population (P13), mostly corresponding to a monocyte-Macrophage pool, which was further characterised. Events from gate P13 were further analysed based on Ly6C and F4/80 markers to classify them as monocytes (P14: Ly6G^hi^ F4/80^lo^) or macrophages (P15: Ly6G^lo^ F4/80^hi^). Alternatively, the P13 population was also analysed based on Ly6C marker in combination with MHC-II to define 4 different populations (P16-19).
