# Supplementary figures and images for: Pro-resolving and anti-arthritic properties of the MC1 selective agonist PL8177
Source: Front Immunol. 2022 Nov 24;13:1078678. doi: 10.3389/fimmu.2022.1078678 (PMC9730523; doi:10.3389/fimmu.2022.1078678)

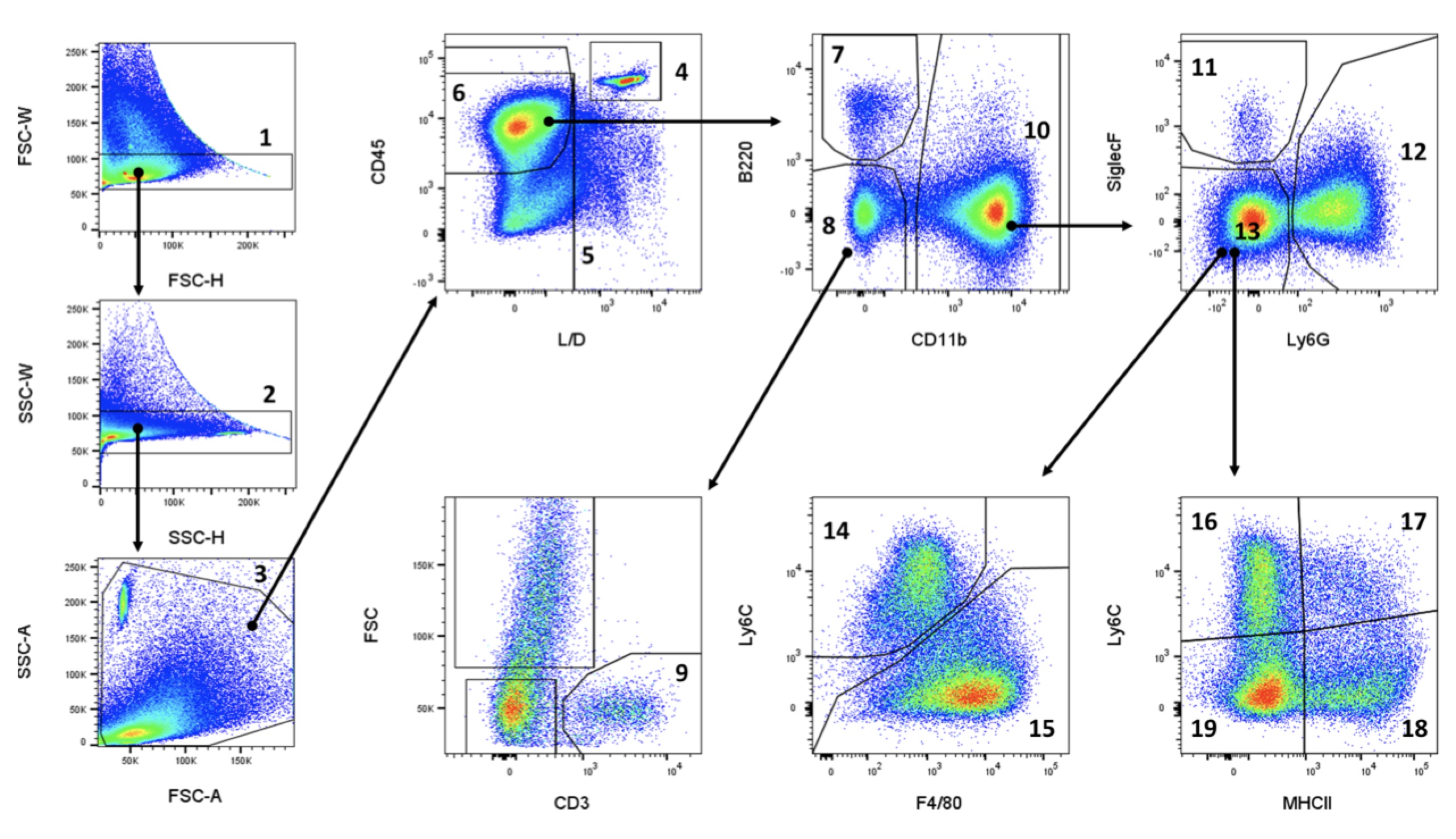

Supplement: Supplementary Figure 1 — Gating strategy for the analysis of hind paw immune cell infiltration. Representative flow cytometry analysis following a sequential gating strategy. Populations of interest are annotated with numbers (populations P1-P19). Recorded events were cleaned to select single events based on forward scatter FSC (P1) and side scatter SSC (P2) signals, and according to cell size (P3). Counting beads (P4) were gated using their autofluorescence and live cells (P5) were selected based on live/dead (L/D) discrimination marker. Live CD45+ immune cells (P6) were categorised in 3 main populations according to the expression of the following markers: B cells (P7: CD45+ CD11b- B220+), T cells (P9: CD3e+ cells selected from P8: CD45+ CD11b- B220-) and myeloid cells (P10: CD45+ B220- CD11b+). Eosinophils (P11: CD45+ CD11b+ SiglecF+) and neutrophils (P12: CD45+ CD11b+ Ly6G+) were selected among myeloid cells based on SiglecF and Ly6G markers, and the double negative myeloid population (P13), mostly corresponding to a monocyte-Macrophage pool, which was further characterised. Events from gate P13 were further analysed based on Ly6C and F4/80 markers to classify them as monocytes (P14: Ly6Ghi F4/80lo) or macrophages (P15: Ly6Glo F4/80hi). Alternatively, the P13 population was also analysed based on Ly6C marker in combination with MHC-II to define 4 different populations (P16-19). [file Image_1.jpeg]
